# 胸膜巨大孤立性纤维瘤合并周围型肺动脉瘤1例

**DOI:** 10.3779/j.issn.1009-3419.2015.08.10

**Published:** 2015-08-20

**Authors:** 远大 程, 阳 高, 位星 张, 春芳 张

**Affiliations:** 410008 长沙，中南大学湘雅医院胸外科 Department of Thoracic Surgery, Xiangya Hospital, Central South University, Changsha 410008, China

**Keywords:** 胸腔, 孤立性纤维瘤, 肺动脉瘤, Toracic cavity, Solitary fbrous tumor, Pulmonary artery aneurysm

## Abstract

胸膜孤立性纤维瘤，临床不多见，约占胸膜肿瘤的5%。肺动脉瘤临床亦不常见，80%位于主肺动脉，周围型较少。胸膜孤立性纤维瘤合并肺动脉瘤临床上尚未见报道。本文报道1例胸腔内巨大孤立性纤维瘤合并周围性肺动脉瘤的患者，其胸腔内的孤立性纤维瘤的生长可能加速了肺动脉瘤的形成和进展。

## Abstract (en)

Solitary fibrous tumor of pleura (SFTP) is uncommon, accounts for less than 5% of all pleural tumors. Pulmonary artery aneurysm (PAA) is also not common, 80% of which often occurs in the main pulmonary trunk and peripheral PAA is rare. SFTP combined with PAA in one patient has not been reported. This paper reports a case of SFTP combined with peripheral PAA, and SFTP maybe accelerate PAA formation.

## 1 临床资料

男性，50岁，2013年1月因胸痛、胸闷就诊，计算机断层扫描（computed tomography, CT）检查发现左侧胸腔占位，左舌叶动静脉畸形（图 1A，图 1B），建议手术，但患者要求保守治疗，予以追踪观察。2014年9月，患者因胸闷、气促加重，再次就诊，CT提示左侧胸腔占位，病变累及肺门，纵隔多发淋巴结肿大，左肺不张，左肺动脉瘤形成，左侧胸腔积液，考虑胸腔种植性转移（图 1C，图 1D）。完善相关检查后，为解除压迫症状，患者强烈要求手术探查。于2014年9月12日行左侧开胸探查术，手术经左侧第5肋床外侧切口进胸，见胸腔内大量淡黄色清亮胸水，左肺压缩不张，胸腔内肿块侵犯左下肺，大小约20 cm，质脆，血运丰富，触之易出血，胸壁、膈肌等多处可见类似病变。经与患者家属沟通后，要求切除病变。因肿瘤血运丰富，无法分块切除，手术直接暴露肺门结构，解剖上、下肺静脉及肺动脉干，切除左全肺，对于胸壁和膈肌的病变行姑息性切除，术中出血约650 mL，输浓缩红细胞2 u。胸腔及胸壁的肿块术后病检结果示：梭形细胞肿瘤，部分细胞有明显异型性，核分裂现象多见，浸润肺组织，结合免疫组化，考虑恶性孤立性纤维瘤。免疫组化结果：CD34（+++）、Bcl-2（+++）、CD99（-）、S100（-）、TTF-1（-）、CK7（-）、CK-Pan（-）、EMA（-）、Vimentin（+）、CR（-）、MC（-）、CK5/6（-）、Ki67（约10%-15%）、TdT（-）、CD20（-）、CD5（-）、CgA（-）、Syn（-）、CD56（-）、F8（-）、CD31（-）。患者术后第10天顺利出院。患者分别于术后1个月（2014年11月）、8个月（2015年5月）再次到我院复查，患者未诉特殊不适，复查胸片示气管左移，左侧呈全肺切除术后状态，右肺纹理及肋膈角清晰（图 2）。

**1:**
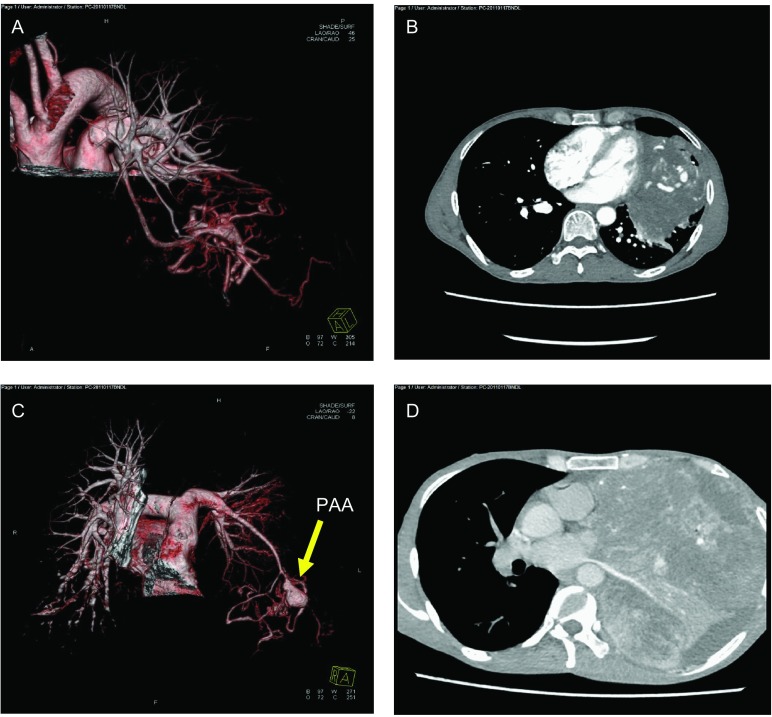
患者影像图片。A和B分别是2013年患者的3D血管成像和增强CT扫描；C和D是2014年的3D血管成像和增强CT扫描。从2014年的C和D图片中，我们可以发现肺动脉瘤已形成，肿块变大，占据左侧胸腔。 Image pictures of patients. A is 3D imaging of vessels and B is enhanced CT scan of chest in 2013; C and D is in 2014. From C and D, we can find PAA has formatted and thoracic mass (SFTP) is larger and full of the left thorax in 2014. PAA: pulmonary artery aneurysm; CT: computed tomography.

**2:**
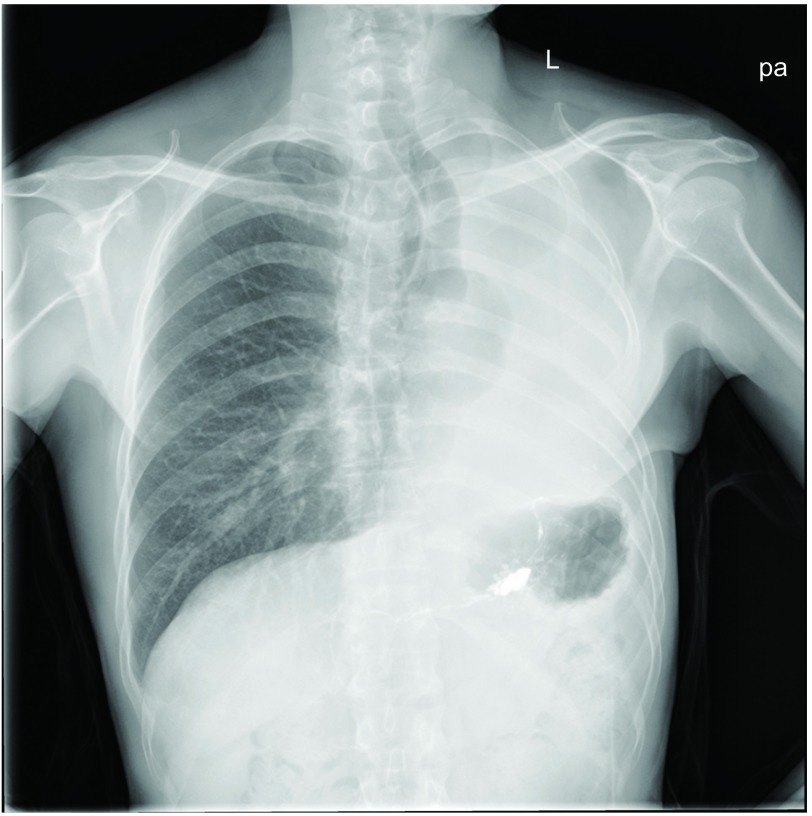
患者术后胸片（2015年5月），显示左全肺切除术后的状态，气管左偏，右肺野很清晰。 Chest X-ray after operation eight months (May in 2015), which shows a state of left pneumonectomy, trachea shift to the left, and right lung field is very clear.

## 2 讨论

胸膜孤立性纤维瘤（solitary fibrous tumors of pleura, SFTP），临床上不常见，占胸膜源性肿瘤的5%，约80%来自脏层胸膜，20%来自壁层胸膜，多见于50岁-70岁患者，无性别差异^[1, 2]^。随着病理学水平的发展，SFTP被认为是起源于间皮下、间充质细胞，免疫组化常提示CD34和bcl-2呈阳性^[3, 4]^。SFTP多为良性，据文献^[5, 6]^报道恶性SFTP约占10%-20%，SFTP对放化疗不敏感，外科完整切除是目前SFTP的主要治疗方式^[7]^，一般的体积不是较大的SFTP，可考虑胸腔镜手术^[8]^，对于体积较大的仍需要开胸，我们曾报道1例巨大SFTP，肿瘤占据左侧整个胸腔，手术完整予以切除^[9]^。对于良性SFTP，其术后10年生存率高达97.5%^[7]^，而恶性SFTP术后有一定的复发率，复发率约30%^[10]^。

肺动脉瘤（pulmonary artery aneurysm, PAA）临床亦非常罕见，尸检中发现其发病率约1/14, 000，发病率与性别、年龄无关，80%位于主肺动脉^[11]^。PAA的发生、发展的机制目前仍不清楚。PAA常分为合并动静脉交通和不合并动静脉交通两大类，另40%见于单纯遗传性毛细血管扩张症，感染因素少；不合并动静脉交通的PAA常见发病原因可能与肺动脉高压、结蹄组织病（如：马凡综合症、Ehlers-Danlos综合征）、感染因素（梅毒、结核）、创伤等因素有关，另外还有一些不明原因的特发性PAA^[12]^。

对于SFTP合并PAA的患者目前尚未见报道，本文报道的左侧胸腔巨大SFTP合并周围性PAA的患者，临床上极为罕见。该患者无肺动脉高压、无明显感染因素、也无明确的外伤史，其肺动脉瘤位于周边，CT提示为合并动静脉畸形，因此考虑可能与遗传因素有关。随着SFTP的生长，瘤体的增大压迫肺组织导致左肺不张，PAA也随之形成而变大。可能的原因为巨大胸腔内肿瘤的压迫，导致肺动脉阻力的增高，肺动脉压力也随着上升所致。

PAA的临床表现无特殊性，主要表现有咯血、胸痛、呼吸困难、咳嗽、发热等，其中严重的咯血是肺动脉的危险信号，常暗示PAA的破裂^[13]^。该患者无明显咯血，主要表现为胸闷、气促，主要是肿瘤的压迫症状。肺血管三维成像是诊断PAA的主要手段之一，但有关PAA治疗，目前尚无统一的治疗指南，外科干预被认为是有效的方法之一。近端的PAA常采用肺动脉成形或置换，周围性的PAA常采用肺叶切除或介入栓塞治疗。本例患者因合并SFTP，且病变累及肺组织和肺门结构，左全肺不张，胸壁和膈肌多处种植转移，因此手术行左全肺切除及胸腔内的减瘤手术。根据患者半年后的随访结果可见，手术到达一定的治疗效果，改善了患者的生活质量。因手术为姑息性切除，且术后的病检结果为恶性SFTP，因此患者术后会存在肿瘤的复发。SFTP目前缺乏有效的放化疗，所以该患者术后需定期复查，必要时可考虑再次手术。

综上可见，该例患者因缺少更早期的临床检查资料，PAA与其胸腔内的SFTP在发病机制上无法判断是否存在相关性，但在后期的发展过程中，随着胸腔内的SFTP的生长，瘤体的变大，可能加速了PAA的形成和进展。但对于胸腔内巨大占位性病变是否是周围性PAA形成的原因之一，尚有待于进一步研究。
